# Prolyl Hydroxylase Domain-Containing Protein 2 (*Phd2*) Regulates Chondrocyte Differentiation and Secondary Ossification in Mice

**DOI:** 10.1038/srep35748

**Published:** 2016-10-24

**Authors:** Shaohong Cheng, Patrick Aghajanian, Sheila Pourteymoor, Catrina Alarcon, Subburaman Mohan

**Affiliations:** 1 Musculoskeletal Disease Center, Veterans Affairs Loma Linda Healthcare System, 11201 Benton Street, Loma Linda, CA 92357, USA; 2 Department of Medicine, Loma Linda University, Loma Linda, CA 92354, USA.

## Abstract

Endochondral ossification plays an important role in the formation of the primary ossification centers (POCs) and secondary ossification centers (SOCs) of mammalian long bones. However, the molecular mechanisms that regulate POC and SOC formation are different. We recently demonstrated that Prolyl Hydroxylase Domain-containing Protein 2 (*Phd2*) is a key mediator of vitamin C effects on bone. We investigated the role of *Phd2* on endochondral ossification of the epiphyses by conditionally deleting the *Phd2* gene in osteoblasts and chondrocytes. We found that the deletion of *Phd2* in osteoblasts did not cause changes in bone parameters in the proximal tibial epiphyses in 5 week old mice. In contrast, deletion of *Phd2* in chondrocytes resulted in increased bone mass and bone formation rate (normalized to tissue volume) in long bone epiphyses, indicating that *Phd2* expressed in chondrocytes, but not osteoblasts, negatively regulates secondary ossification of epiphyses. *Phd2* deletion in chondrocytes elevated mRNA expression of hypoxia-inducible factor (HIF) signaling molecules including *Hif-1α*, *Hif-2α*, *Vegfa*, *Vegfb*, and *Epo*, as well as markers for chondrocyte hypertrophy and mineralization such as *Col10*, *osterix*, alkaline phosphatase, and bone sialoprotein. These data suggest that *Phd2* expressed in chondrocytes inhibits endochondral ossification at the epiphysis by suppressing HIF signaling pathways.

Endochondral ossification plays an important role in the development of the primary ossification centers (POCs) and secondary ossification centers (SOCs) of mammalian long bones^1^,^2^. The formation of POCs has been well characterized and is known to begin with the condensation of mesenchymal cells which differentiate into *Col2*-expressing chondrocytes and form the epiphyseal cartilage plate (growth plate) surrounded by the perichondrium. Osteoprogenitor cells from the perichondrium differentiate into osteoblasts and form the lateral bone collar which becomes the cortical bone. The growth plate chondrocytes proliferate and undergo a differentiation process called hypertrophy marked by *Col10* expression. These hypertrophic chondrocytes begin to mineralize the extracellular matrix and form the primary spongiosa. The ossified cartilage is further remodeled by osteoblasts and osteoclasts brought in by vascular invasion^1^,^3^,^4^.

While the cellular and molecular events associated with the formation of POCs have been well studied, the underlying molecular mechanisms involved in the formation of the SOCs are poorly understood. Similar to POC formation, SOC formation also involves chondrocyte hypertrophy and mineralization of the extracellular matrix of chondrocytes. However, there are several major differences between the two processes. Firstly, while the POC forms at embryonic day 15.5 (E15.5), SOC formation does not begin until postnatal days 5 to 7 in rodents^5^,^6^. Secondly, endochondral ossification in the POCs progresses in a single direction from the growth plate towards the mid-diaphysis, while endochondral ossification in the SOCs starts from the cartilaginous center of the epiphysis and radially progresses outwards until reaching the apical articular cartilage and the basal growth plate. Thirdly, SOC formation involves the formation of a cartilage canal to provide a cell source for the SOC development^7^,^8^,^9^. Hence, the initial formation of SOCs, which occurs around week 1 to 3 in rodents, relies heavily on chondrocytes rather than on both osteoblasts and chondrocytes as in the case of POC formation. The molecular pathways that regulate various cellular processes that occur during SOC formation are poorly understood.

Endochondral and intramembranous ossification are regulated by a family of genes named Prolyl Hydroxylase Domain-containing Proteins (Phds) which include *Phd1*, *Phd2*, and *Phd3*^10^. PHDs contain highly conserved hydroxylase domains in the catalytic carboxy-terminals and are important regulators of hypoxia-inducible factors (HIFs)^10^. We have previously discovered that *Phd2* was abundantly expressed in osteoblasts and chondrocytes, and *Phd2* played different roles in osteoblasts and chondrocytes during the formation of POCs^11^,^12^. *Phd2* promotes osteoblast differentiation by up-regulating osterix (*Osx*) expression independent of HIF signaling, and deletion of *Phd2* in osteoblasts reduced bone size and trabecular bone mass in POCs due to a reduced bone formation rate^11^. However, *Phd2* suppresses chondrocyte differentiation by inhibiting the HIF signaling pathway, and the disruption of *Phd2* in chondrocytes resulted in increased trabecular bone mass and cortical thickness in POCs^12^. Hence, *Phd2* plays distinct roles in osteoblasts versus chondrocytes.

Since SOC formation appears to differ from POC formation in several aspects and since *Phd2* exerts different roles in osteoblasts versus chondrocytes during POC formation, we asked whether *Phd2* also affects trabecular bone mass in the long bone epiphysis, and if so, if the effect of *Phd2* on SOC formation is different in the two types of cells. To address these questions, we conditionally disrupted the *Phd2* gene in osteoblasts and chondrocytes and compared bone phenotypes as well as the expression of markers for chondrocyte hypertrophy and mineralization in SOCs of epiphyses. We found interesting differences between the two conditional knockout phenotypes which are consistent with the idea that different mechanisms regulate POC and SOC formation.

## Results

### Conditional deletion of *Phd2* in osteoblasts and chondrocytes

We used *Col1α2-iCre* transgenic mice to disrupt *Phd2* expression in osteoblasts^13^,^14^. We have previously used this Cre line to delete *Phd2* in osteoblast lineage cells, and revealed predominant expression of iCre in bone tissues and a specific deletion of *Phd2* in osteoblasts by iCre activity detected by western blot analysis^11^,^15^. The epiphyseal bone phenotypes of the *Phd2*^*Col1-iCre*^ mice and their corresponding control littermates were analyzed. To disrupt *Phd2* expression in chondrocytes, we used the *Col2α1-Cre* transgenic mice^16^. Ovchinnikov *et al.* detected *Col2α1-Cre* activity in all cartilaginous primordia of the developing bones. In bones, *Col2α1-Cre* activity was detected specifically in chondrocytes but not in osteoblasts^16^. We have also previously demonstrated the specific deletion of *Phd2* by *Col2α1-Cre* in chondrocytes and the specificity of this mouse line has been well documented in many studies^12^,^17^,^18^,^19^,^20^. The epiphyseal bone phenotypes of the *Phd2*^*Col2-Cre*^ mice and their corresponding control littermates were analyzed.

The formation of the SOC begins around postnatal day 7 in mice and the ossification of the epiphyseal cartilage is nearly complete around week 3 in mice^6^. The vascularization of distal femoral and proximal tibial epiphyses of mice begins around week 2 to provide a source of cells for endochondral ossification^21^,^22^,^23^. In our previous studies, we did not detect any gender-genotype interactions in the *Phd2*^*Col1-iCre*^ and *Phd2*^*Col2-Cre*^ mice for bone parameters at multiple skeletal sites including the total body, femur, tibia, and vertebrae^11^,^12^. In this study, we also tested whether a lack of *Phd2* in chondrocytes affects SOC formation in a gender-dependent manner and found no gender-genotype interaction on trabecular parameters in the femoral epiphysis of the *Phd2*^*Col2-Cre*^ and control mice (data not shown). Therefore, we performed phenotypical analyses with mixed genders.

### *Phd2* ^*Col2-Cre*^ but not *Phd2* ^*Col1-iCre*^ mice exhibited increased bone mass in SOCs of long bone epiphyses

**Figure 1:**
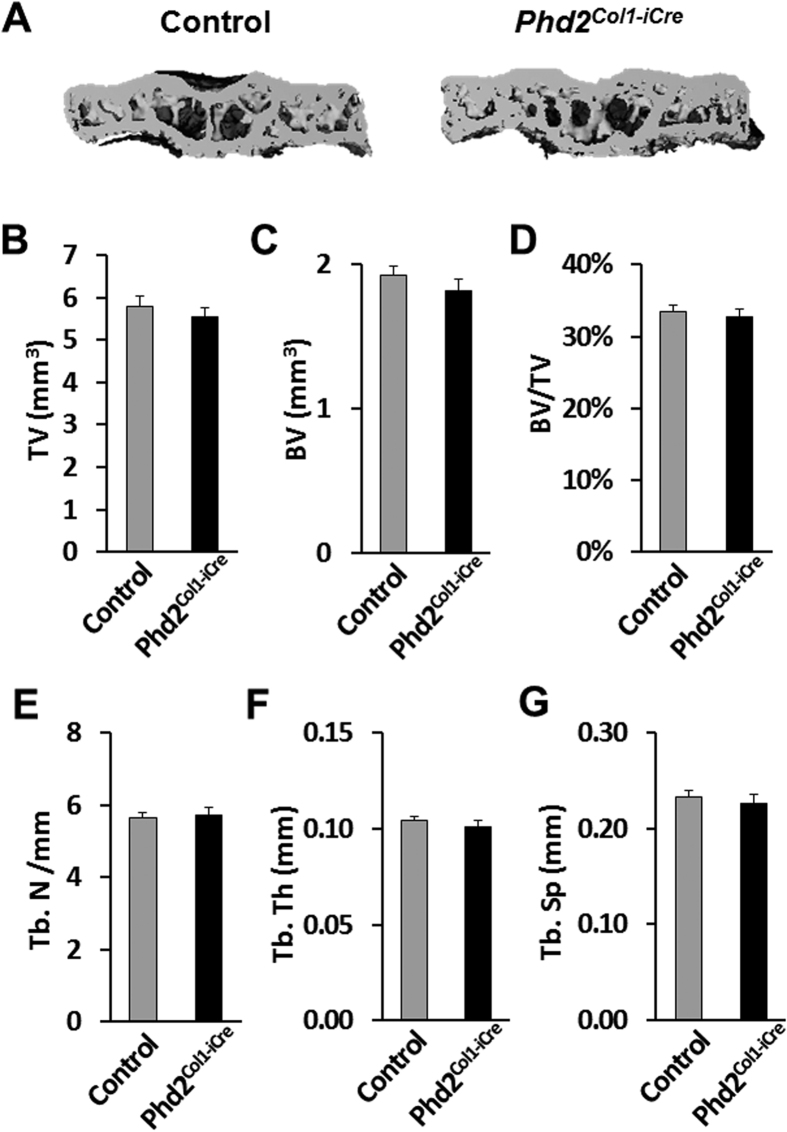
Micro-computed tomography (μCT) analysis revealed no significant changes in bone parameters in the proximal tibial epiphyses of the osteoblast-specific *Phd2* knockout mice. (**A**) Representative 3D images of the proximal tibial epiphyses of 5 week old control and *Phd2*^*Col1-iCre*^ mice. (**B–G**) Quantitative data of TV, BV, BV/TV, Tb. N, Tb. Th, and Tb. Sp of the proximal tibial epiphyses in control and *Phd2*^*Col1-iCr*^ mice. BV, bone volume; TV, total volume; Tb. N, trabecular number; Tb. Th, trabecular thickness; Tb. Sp, trabecular separation. *n* = 5/group. Data were presented as mean ± SEM.

Micro-computed tomography (μCT) analyses of proximal tibial epiphyses in 5 week old *Phd2*^*Col1-iCre*^ mice and corresponding control littermates revealed no significant differences in bone parameters between the two genotypes (Fig. 1). Figure 1A shows representative 3D images of the proximal tibial epiphyses of the control and *Phd2*^*Col1-iCre*^ mice. All bone parameters, including total volume (TV), bone volume (BV), BV/TV, trabecular number (Tb. N), trabecular thickness (Tb. Th), and trabecular separation (Tb. Sp), were unaltered in the *Phd2*^*Col1-iCre*^ mice compared to control mice (Fig. 1B–G). In contrast to the lack of SOC phenotype in the tibial epiphyses of *Phd2*^*Col1-iCre*^ mice, we previously found that *Phd2* expressed in osteoblasts influenced POC formation as reflected by the osteopenia phenotype in the secondary spongiosa of long bones of *Phd2*^*Col1-iCre*^ mice at 5 weeks of age^11^.

**Figure 2:**
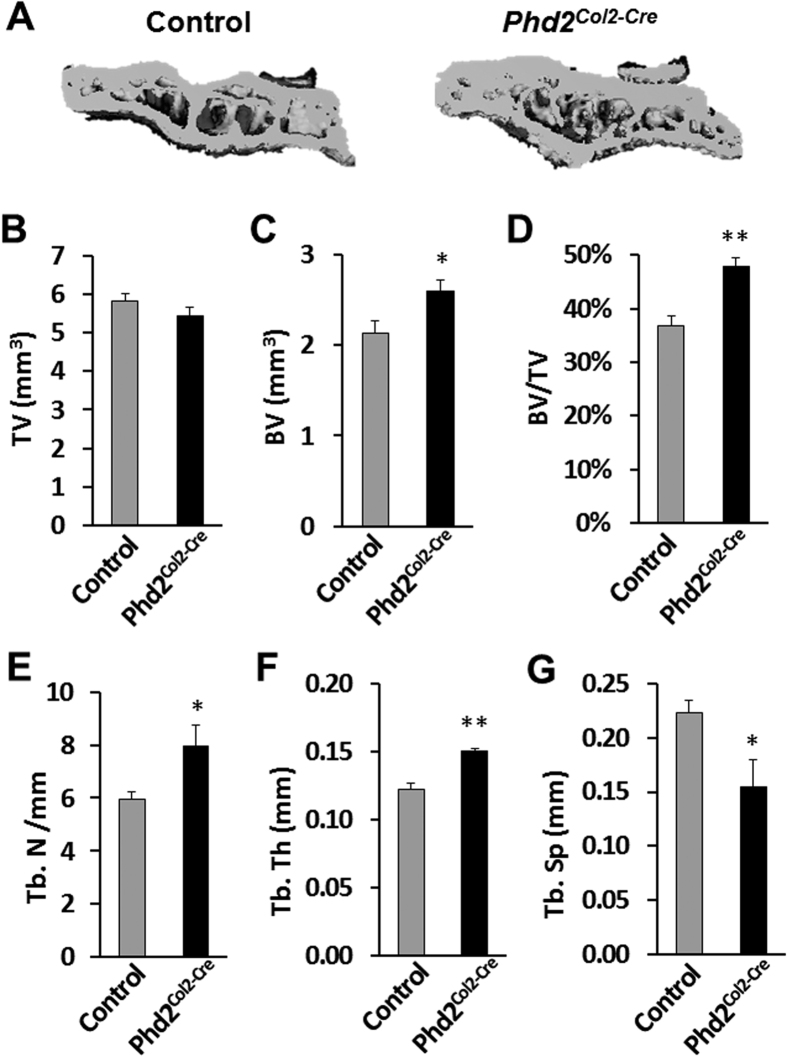
Micro-CT analysis revealed increased bone mass in the proximal tibial epiphyses of the chondrocyte-specific *Phd2* knockout mice. (**A**) Representative 3D images of the proximal tibial epiphyses of 5 week old control and *Phd2*^*Col2-Cre*^ mice. (**B–G**) Quantitative data of TV, BV, BV/TV, Tb. N, Tb. Th and Tb. Sp of the proximal tibial epiphyses in control and *Phd2*^*Col2-Cre*^ mice. BV and BV/TV were significantly increased in the *Phd2*^*Col2-Cre*^ mice compared to controls. Tb. N and Tb. Th were also increased while Tb. Sp was decreased in the *Phd2*^*Col2-Cre*^ mice compared to controls. *P < 0.05, **P < 0.01, *n* = 5/group. Data were presented as mean ± SEM.

**Figure 3:**
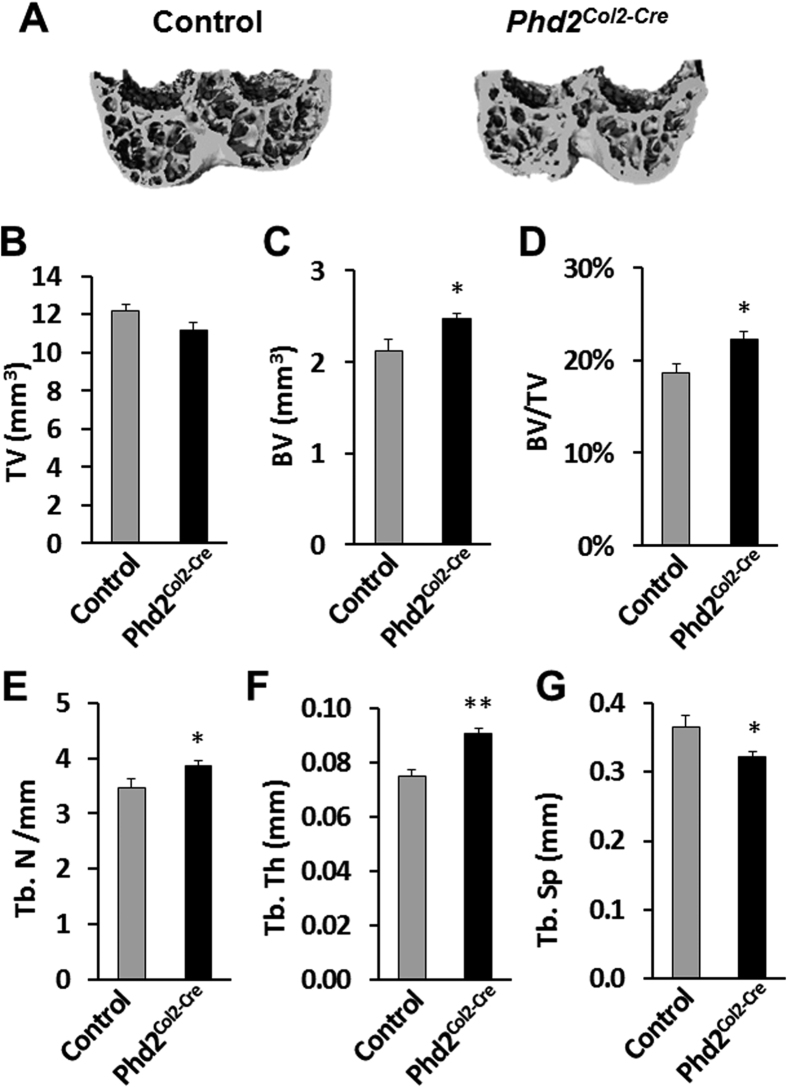
Micro-CT analysis revealed increased bone mass in the distal femoral epiphyses of the chondrocyte-specific *Phd2* knockout mice. (**A**) Representative 3D images of the distal femoral epiphyses of 4 week old control and *Phd2*^*Col2-Cre*^ mice. (**B–G**) Quantitative data of TV, BV, BV/TV, Tb. N, Tb. Th and Tb. Sp of the distal femur epiphyses in control and *Phd2*^*Col2-Cre*^ mice. BV and BV/TV were significantly increased in the *Phd2*^*Col2-Cre*^ mice compared to controls. Tb. N and Tb. Th were also increased while Tb. Sp was decreased in the *Phd2*^*Col2-Cre*^ mice compared to controls. *P < 0.05, **P < 0.01, *n* = 8/group. Data were presented as the mean ± SEM.

In sharp contrast, we observed significantly increased bone mass in the *Phd2*^*Col2-Cre*^ mice at the proximal tibial epiphyses compared to littermate controls (Fig. 2). Figure 2A shows representative 3D images of the proximal tibial epiphyses of control and *Phd2*^*Col2-Cre*^ mice. While tissue volume was not changed in the *Phd2*^*Col2-Cre*^ mice (Fig. 2B), bone volume and bone volume/tissue volume were increased by 21% (*P* < 0.05) and 30% (*P* < 0.01), respectively, in the *Phd2*^*Col2-Cre*^ mice compared to controls (Fig. 2C,D). Trabecular number and thickness were increased by 33% (*P* < 0.05) and 24% (*P* < 0.01), respectively, in the *Phd2*^*Col2-Cre*^ mice compared to controls (Fig. 2E,F). In contrast, trabecular separation was decreased by 31% (*P* < 0.05) in the *Phd2*^*Col2-Cre*^ mice compared to controls (Fig. 2G). Increased endochondral ossification was further confirmed in the SOCs of the distal femoral epiphyses of the *Phd2*^*Col2-Cre*^ mice (Fig. 3). Similarly, increases in bone volume (17%, *P* < 0.05), bone volume/tissue volume (20%, *P* < 0.05), trabecular number (12%, *P* < 0.05), and thickness (21%, *P* < 0.01) and a decrease in trabecular separation (11%, *P* < 0.05) were detected in the SOCs of the distal femoral epiphyses of the *Phd2*^*Col2-Cre*^ mice compared to controls (Fig. 3).

### *Phd2* deletion in chondrocytes increased bone formation rate but normal resorption in SOCs of long bone epiphyses in the *Phd2* ^*Col2-Cre*^mice

**Figure 4:**
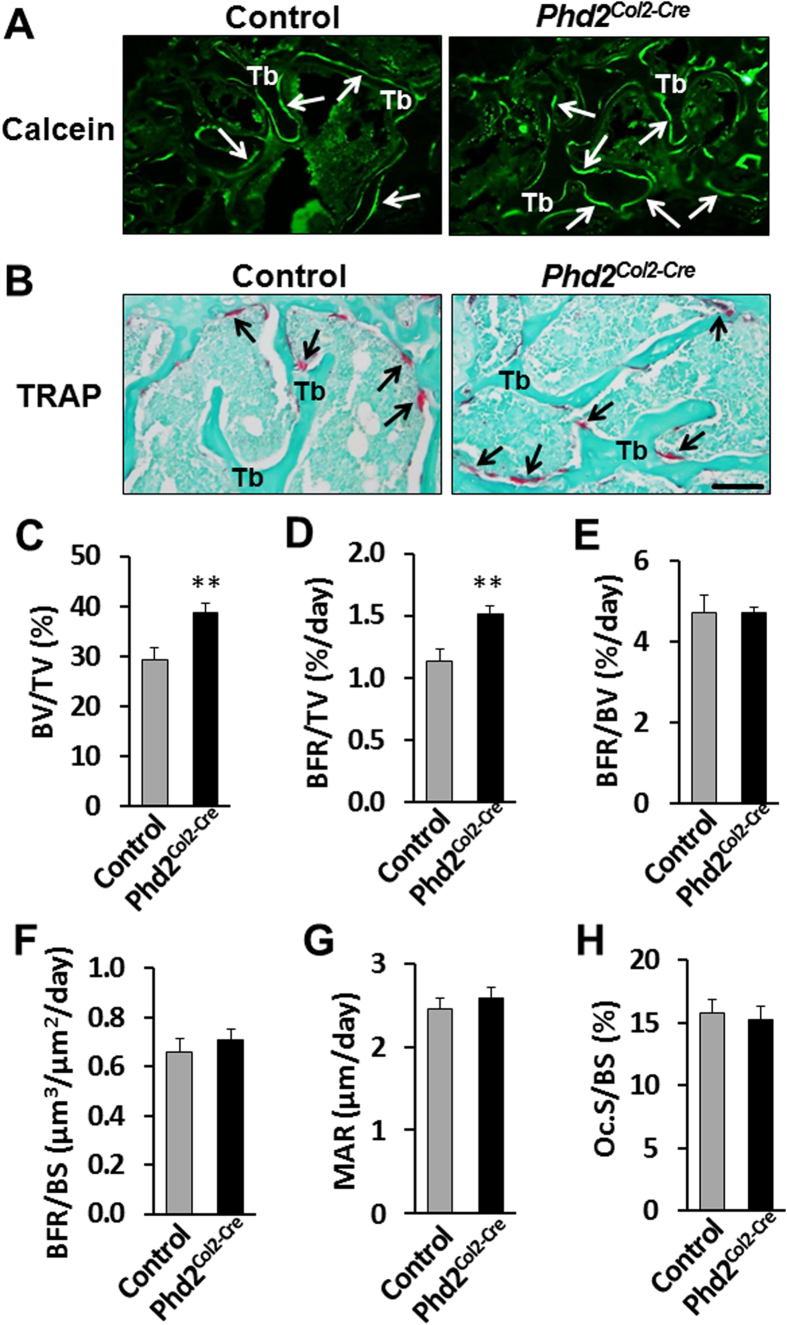
Histomorphometry analysis revealed increased bone formation rate in the epiphyses of the chondrocyte-specific *Phd2* knockout mice. (**A**) Calcein labeling of trabeculae of the femoral epiphyses of the 4 week old control and *Phd2*^*Col2-Cre*^ mice. Arrows show the calcein labeling. (**B**) TRAP staining of trabeculae of the tibial epiphyses of the 4 week old control and *Phd2*^*Col2-Cre*^ mice. Arrows show the TRAP positive bone surface. (**C–H**) BV/TV, BFR/TV, BFR/BV, BFR/BS, MAR, and Oc.S/BS of the proximal tibial epiphyses in the control and *Phd2*^*Col2-Cre*^ mice. TRAP, tartrate-resistant acid phosphatase; Tb, trabecular bone; BFR, bone formation rate; BS, bone surface; MAR, mineral apposition rate; Oc.S, osteoclast surface. *P < 0.05, **P < 0.01, *n* = 9/group. Data were presented as mean ± SEM. Bar = 50 μM.

Since we detected increased bone mass in SOCs of *Phd2*^*Col2-Cre*^ mice, we next examined bone formation and resorption rates in these mice. Figure 4A,B show calcein labeling and tartrate-resistant acid phosphatase (TRAP) staining in SOCs of control and *Phd2*^*Col2-Cre*^mice (arrows show the calcein labeling and TRAP signal). Histomorphometric analyses revealed a 31% increase of bone volume to tissue volume in the tibial epiphyses of the *Phd2*^*Col2-Cre*^ mice compared to control mice (*P* < 0.01, Fig. 4C), a finding consistent with the μCT data shown in Figs 2D and 3D. Bone formation rate (BFR) using different referents such as tissue volume, bone volume, and bone surface have been shown to measure different bone formation properties^24^,^25^. While BFR adjusted for tissue volume was increased by 34% in the *Phd2*^*Col2-Cre*^ mice (*P* < 0.01, Fig. 4D), changes in BFR using bone volume and bone surface as referents were not significant in the *Phd2*^*Col2-Cre*^ mice compared to controls (Fig. 4E,F). Mineral apposition rate (MAR) and osteoclast surface to BS (Oc.S/BS) were also not significantly altered in the *Phd2*^*Col2-Cre*^ mice compared to control mice (Fig. 4G,H).

### Elevated marker expression for chondrocyte hypertrophy, mineralization, and HIF signaling in the epiphyses of the *Phd2* ^*Col2-Cre*^ mice

**Figure 5:**
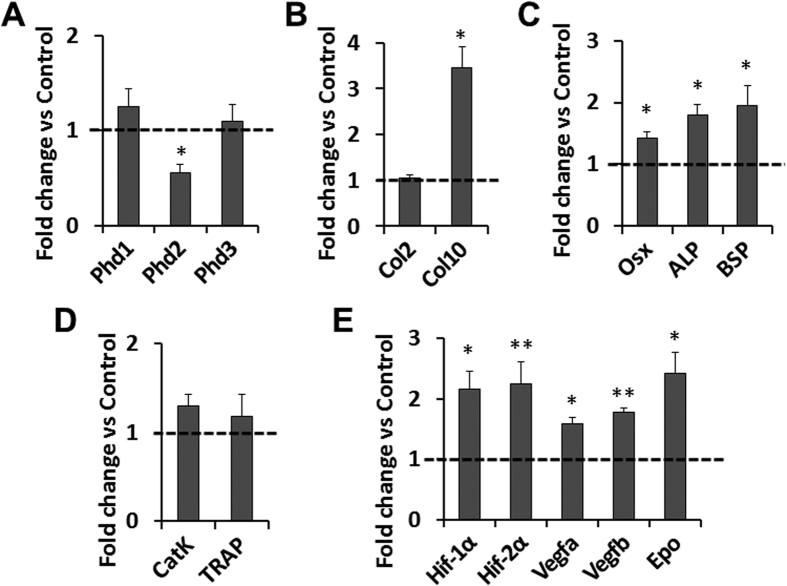
Elevated expression of chondrocyte hypertrophy and mineralization markers and HIF signaling in the secondary ossification center (SOC) of the 4 week old chondrocyte-specific *Phd2* knockout mice. (**A**) Real time RT-PCR revealed that *Phd2* mRNA level was decreased by 44% in the SOCs of the chondrocyte-specific knockout mice, while *Phd1* and *Phd3* mRNA levels were not significantly changed in the knockouts. (**B**) mRNA level of *Col2* and *Col10* in the SOCs of the *Phd2*^*Col2-Cre*^ mice. (**C**) Expression of *Osx*, ALP and BSP in the SOCs of the *Phd2*^*Col2-Cre*^ mice. (**D**) Expression of bone resorption markers, CatK and TRAP, in the SOCs of the *Phd2*^*Col2-Cre*^ mice. (**E**) *Hif-1α* and *Hif-2α* mRNA levels were increased by 2-fold, and expression of HIF targets, *Vegfa*, *Vegfb*, and *Epo,* were also increased in the SOCs of the *Phd2*^*Col2-Cre*^ mice. *P < 0.05, **P < 0.01, *n* = 7/group. Data were normalized to controls and presented as mean ± SEM.

To further investigate the mechanisms for increased endochondral bone formation in the epiphyses of the *Phd2*^*Col2-Cre*^ mice, we isolated the proximal tibial epiphyses of 4 week old control and *Phd2*^*Col2-Cre*^ mice for RNA extraction and gene expression assessments. First, we measured expression levels of all three Phds. We detected a 44% reduction of *Phd2* mRNA levels in the epiphyses of the *Phd2*^*Col2-Cre*^ mice compared to control mice (*P* < 0.05, Fig. 5A). The deletion of *Phd2* in the epiphysis was not 100% because, in addition to chondrocytes, the tibial epiphysis contains other cell types such as myelopoietic and erythropoietic cells. Furthermore, penetration of Col2α1-Cre mediated gene ablation is about 95% in chondrocytes^16^. In contrast to the sharp reduction of the *Phd2* mRNA level, *Phd1* and *Phd3* mRNA levels were not altered in the *Phd2*^*Col2-Cre*^ mice compared to the controls (Fig. 5A). Next, we examined the expression levels of *Col2* and *Col10*, markers of proliferating and differentiating chondrocytes, respectively. While expression of *Col2* was not altered in the epiphyses of the *Phd2*^*Col2-Cre*^ mice, *Col10* expression was increased by 245% (*P* < 0.05, Fig. 5B). The increased expression of *Col10* in the epiphyses of *Phd2*^*Col2-Cre*^ mice indicates increased chondrocyte hypertrophy in the SOCs of the *Phd2*^*Col2-Cre*^ mice. *Osx* expression is also stimulated during chondrocyte differentiation and expressed in pre-hypertrophic and hypertrophic chondrocytes^26^,^27^,^28^. We observed a 43% (*P* < 0.05) increase in the *Osx* mRNA level in the *Phd2*^*Col2-Cre*^ mice (Fig. 5C). We also detected increased expression of mineralization markers, alkaline phosphatase (ALP) and bone sialoprotein (BSP) by 80% and 95%, respectively, in the *Phd2*^*Col2-Cre*^ mice compared to controls (*P* < 0.05, Fig. 5C). In contrast, expression levels of bone resorption markers, CatK and TRAP, were not altered in the *Phd2*^*Col2-Cre*^ mice compared to controls (Fig. 5D).

We have previously reported that chondrocyte-specific deletion of *Phd2* up-regulated HIF signaling in the growth plate chondrocytes^12^. Consistently, we also found that *Hif-1α* and *Hif-2α* mRNA levels were increased by 116% (*P* < 0.05) and 125% (*P* < 0.01), respectively, in the epiphyses of the *Phd2*^*Col2-Cre*^ mice compared to controls (Fig. 5E). The expression levels of HIF signaling targets, *Vegfa*, *Vegfb*, and *Epo*, were increased by 59% (*P* < 0.05), 78% (*P* < 0.01), and 141% (*P* < 0.05), respectively, in the epiphyses of the *Phd2*^*Col2-Cre*^ mice compared to controls (Fig. 5E).

### Elevated marker expression for chondrocyte hypertrophy and HIF signaling in primary chondrocytes treated with PHD inhibitor DMOG

**Figure 6:**
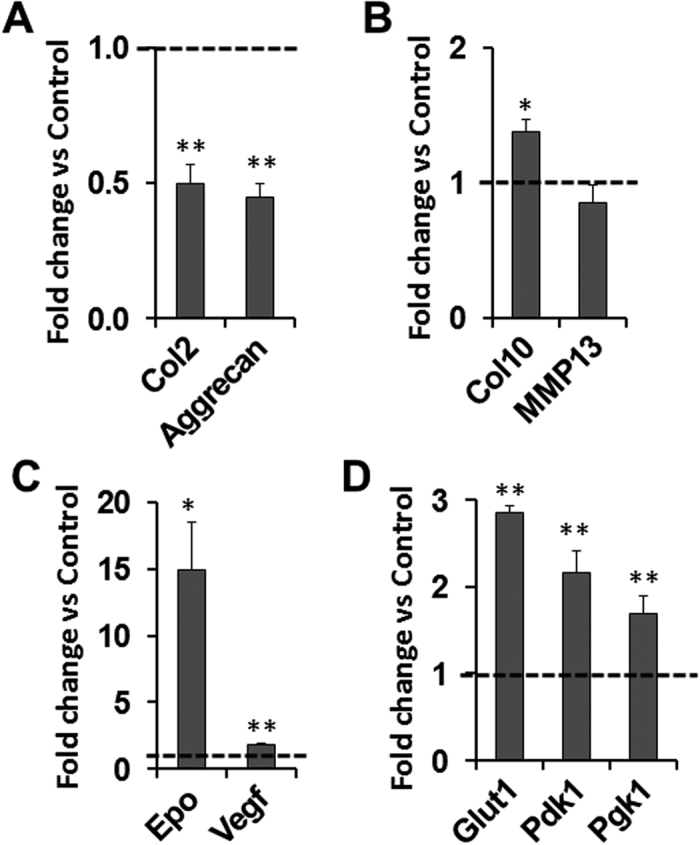
Elevated expression of chondrocyte hypertrophy marker and HIF signaling targets in SOC primary chondrocytes treated with PHD inhibitor DMOG. Primary chondrocytes isolated from SOCs were treated with 500 μM PHD inhibitor DMOG or vehicle control. Cells were extracted for real time RT-PCR analysis. (**A**) mRNA levels of *Col2* and Aggrecan in DMOG treated chondrocytes (normalized to vehicle control). (**B**) mRNA levels of hypertrophy markers, *Col10* and MMP13, in DMOG treated chondrocytes. (**C**) mRNA levels of HIF signaling targets, *Epo* and *Vegf*, in DMOG treated chondrocytes. (**D**) mRNA levels of HIF signaling targets, *Glut1*, *Pdk1*, and *Pgk1*, in DMOG treated chondrocytes. *P < 0.05, **P < 0.01, *n* = 4/group. Data were normalized to controls and presented as mean ± SEM.

We further tested the regulation of HIF signaling by *Phd2* in cultured primary chondrocytes isolated from SOCs of the epiphyses. The primary chondrocytes were treated with 500 μM DMOG, a PHD inhibitor, or vehicle control. We found that mRNA levels of chondrocyte markers, *Col2* and Aggrecan, were reduced about 50% (*P* < 0.01) in cells treated the DMOG compared to vehicle controls (Fig. 6A). The mRNA level of chondrocyte hypertrophy marker *Col10* was increased by 38% (*P* < 0.05) while MMP13 expression was unaltered in the DMOG treated cells (Fig. 6B). Treatment of DMOG also elevated HIF signaling targets. *Epo* mRNA level was increased by 15-fold (*P* < 0.05) and *Vegf* expression was increased by 86% (*P* < 0.01) in the DMOG treated SOC chondrocytes (Fig. 6C). HIF signaling also affects glycolytic metabolism^29^,^30^,^31^. We found the mRNA levels of glycolytic enzymes, *Glut1*, *Pdk1*, and *Pgk1*, were also up-regulated by 184%, 115%, and 68%, respectively, in the DMOG treated cells compared to control cells (Fig. 6D). These data are consistent with the up-regulation of HIF signaling and promotion of chondrocyte hypertrophy seen in epiphyseal chondrocytes of *Phd2*^*Col2-Cre*^ mice (Fig. 5).

## Discussion

In this study, we investigated the roles of *Phd2* in osteoblasts and chondrocytes in the formation of SOCs in the long bone epiphyses using cell type-specific knockout mouse models. We found that conditional deletion of *Phd2* in osteoblasts using the *Phd2*^*Col1-iCre*^ transgenic mice had no significant effect on bone parameters in the proximal tibial epiphyses at 5 weeks of age. In contrast, conditional deletion of *Phd2* in chondrocytes using the *Phd2*^*Col2-Cre*^ transgenic mice increased trabecular bone mass in the long bone epiphyses, thus demonstrating a negative role for *Phd2* expressed in chondrocytes in regulating endochondral ossification. We found that the increased trabecular bone mass in SOCs of *Phd2*^*Col2-Cre*^ mice was due to increased bone formation and not due to reduced bone resorption. *Phd2* has no effect on osteoclast activity. We further demonstrated elevated HIF signaling and expression of hypertrophy and mineralization markers including *Col10*, *Osx*, ALP, BSP in the SOCs of the *Phd2*^*Col2-Cre*^ mice, which likely contributed to the increased ossification of the SOCs in the *Phd2*^*Col2-Cre*^ mice. The *Phd2* regulation of HIF signaling and chondrocyte hypertrophy was further confirmed in primary SOC chondrocytes by treating with PHD inhibitor.

We have previously reported the bone phenotype in the diaphyseal and metaphyseal regions of long bones in the osteoblast and chondrocyte-specific *Phd2* knockout mice. The development and elongation of the diaphysis initiated from the formation of POCs in long bones. Since both osteoblasts and chondrocytes are involved in the endochondral ossification of the POC, knocking out of *Phd2* in either osteoblasts or chondrocytes yielded bone phenotypes, though with distinct difference. In osteoblasts, *Phd2* acts as a positive regulator for osteoblast differentiation and bone formation, while in chondrocytes, *Phd2* exerts negative effects on chondrocyte differentiation and endochondral ossification^11^,^12^. Therefore, we observed an osteopenia phenotype in the long bone diaphyses of the osteoblast-specific *Phd2* knockout mice but increased bone mass in the long bone diaphyses of the chondrocyte-specific *Phd2* knockout mice^11^,^12^. This is in contrast to the phenotypes in the SOCs of long bone epiphyses in these knockout mice. These data suggest that *Phd2* expressed in chondrocytes exerts an important effect on trabecular bone mass during embryonic and prepubertal growth periods in the epiphysis. However, *Phd2* expressed in osteoblasts appears to be inconsequential for epiphyseal bone formation during these growth periods. The SOC is also vascularized in adult mice, thus raising the possibility of vascular supply of osteoblast and osteoclast precursors during endochondral bone formation at the epiphysis^32^,^33^; however, at 4–5 weeks of age when the SOC is newly formed, the impact of the osteoblast precursors derived from the circulation on endochondral ossification seems minimal.

The HIF-mediated signaling pathway plays an important role in the maintenance and differentiation of chondrocytes in the hypoxic cartilage, and *Phd2* is the major prolyl hydroxylase targeting HIF proteins^34^,^35^,^36^. We have previously reported that deletion of *Phd2* stimulated the HIF signaling pathway during endochondral bone formation of POCs^12^. In this study, we have also observed elevated HIF signaling in the epiphyses of chondrocyte-specific *Phd2* knockout mice as well as increased expression of hypertrophy and mineralization markers such as *Col10*, *Osx*, ALP, and BSP. Mechanistically similar regulation of chondrocyte hypertrophy and function by Phd2-HIF signaling was seen in both POC and SOC formation, further confirming an inhibitory role for *Phd2* in chondrocyte differentiation and endochondral bone formation.

**Figure 7:**
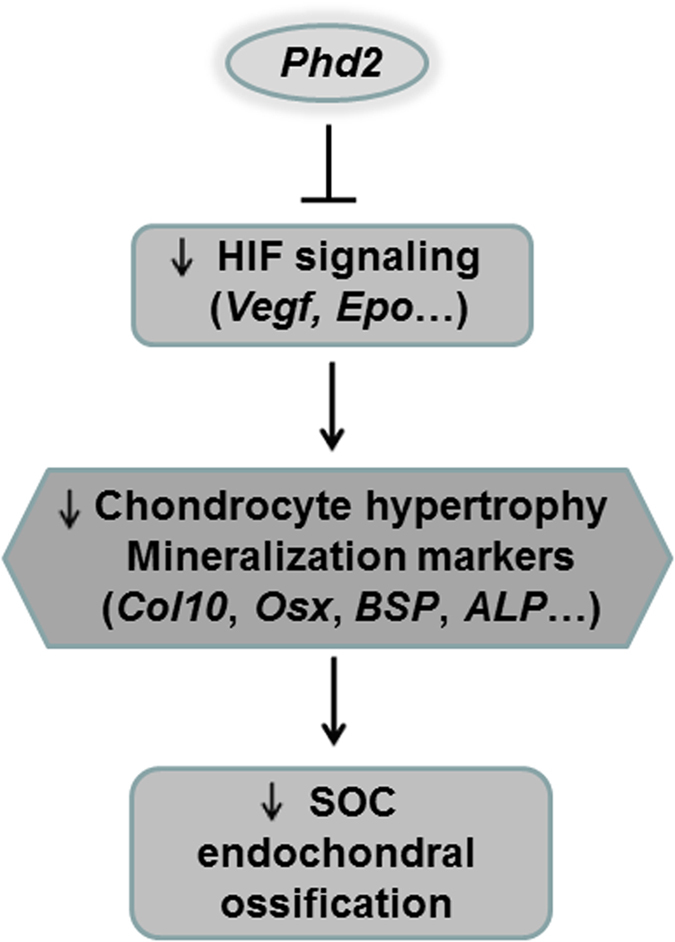
Model of *Phd2* function in chondrocytes during SOC formation. *Phd2* plays a distinct role in chondrocytes in the development of SOCs at the epiphyses of long bones. In chondrocytes, *Phd2* mediates the degradation of HIFs and down regulates HIF signaling. This further down regulates the expression of genes required for chondrocyte hypertrophy and mineralization, such as *Col10*, *Osx*, BSP, and ALP, and inhibits endochondral ossification. *Phd2* expressed in chondrocytes is a negative regulator of endochondral ossification.

Thus, the contrasting phenotypes in the long bone epiphyses of *Phd2*^*Col1-iCre*^ and *Phd2*^*Col2-Cre*^ mice indicate different roles for *Phd2* in osteoblasts versus chondrocytes during endochondral ossification at the epiphyses. Based on our present data and previously published data, we propose a model for the role of *Phd2* in the formation of SOCs (Fig. 7). Knockout of *Phd2* gene in chondrocytes promotes HIF signaling and increases bone mass, demonstrating a negative role for *Phd2* expressed in chondrocytes in regulating endochondral ossification at the epiphysis. The importance of HIF signaling in regulating chondrocyte differentiation has been well established. Thus, in chondrocytes, *Phd2* inhibits HIF signaling including *Hif-1α, Hif-2α*, and HIF downstream targets, *Vegfa, Vegfb,* and *Epo*. This down-regulation of HIF signaling further inhibits chondrocyte differentiation and down-regulates expression of hypertrophy markers such as *Col10*, as well as mineralization markers including *Osx*, BSP, and ALP, thus impeding endochondral ossification at the epiphyses (Fig. 7).

In previous studies, we have determined that *Phd2* is the most abundant Phd isoform expressed in bone cells^37^. While DMOG has been widely used in the literature to inhibit *Phd2* activity, further studies involving knockdown of *Phd2* using *Phd2*-specific shRNA are required to confirm our *in vitro* findings.

POC formation is known to be regulated by a number of growth factors such as PTHrP, Ihh, IGF-1, Wnts and BMPs. Since SOC formation differs from POC formation, whether or not SOC formation also involves these factors needs to be elucidated. Our laboratory has recently discovered that thyroid hormone (TH) played a key role in the formation of the SOC since SOC formation coincides with the time when peak TH levels are attained. Accordingly, TH deficient mice exhibited severely comprised SOC development^6^. Furthermore, TH promoted SOC ossification by activating Ihh and Osx signaling^6^; however, the relationship between TH and other signaling pathways needs to be investigated.

## Methods

### Animals

To generate osteoblast-specific *Phd2* knockout mice, *Phd2* floxed mice (*Phd2*^*flox/flox*^) were crossed with the *Col1α2-iCre* transgenic line which expresses improved Cre recombinase (iCre) in *Col1α2*-expressing cells^13^,^14^. The *Phd2*^*flox/flox*^ mice were first bred to the *Col1α2-iCre* mice to generate the *Phd2*^*flox/+*^;*Col1α2-iCre* mice. The *Phd2*^*flox/+*^;*Col1α2-iCre* mice were then backcrossed with *Phd2*^*flox/flox*^ mice to generate *Phd2*^*flox/flox*^;*Col1α2-iCre*, the osteoblast-specific *Phd2* knockout mice (*Phd2*^*Col1-iCre*^), and the corresponding littermate controls. Similarly, to generate chondrocyte-specific *Phd2* knockout mice, the *Phd2*^*flox/flox*^ mice were first bred to the *Col2α1-Cre* mice to generate *Col2α1-Cre*;*Phd2*^*flox/+*^ mice^16^. The *Col2α1-Cre*;*Phd2*^*flox/+*^ mice were then backcrossed with *Phd2*^*flox/flox*^ mice to generate *Phd2*^*flox/flox*^;*Col2α1-Cre*, the chondrocyte-specific *Phd2* knockout mice (*Phd2*^*Col2-Cre*^), and the corresponding littermate controls. The genetic background of all these mouse lines is C57BL/6.

Animals were housed in the VMU at VA Loma Linda Healthcare System (Loma Linda, CA) under standard approved laboratory conditions. Animal procedures were performed according to protocols approved by the Institutional Animal Care and Use Committee of the VA Loma Linda Healthcare System. Isoflurane was used for anesthesia, and CO_2_ exposure was used for euthanasia followed by cervical dislocation.

### Micro-computed tomography analysis

Conditional knockout and control mice of 4 to 5 week old were sacrificed, and the legs were fixed for 5 days in 10% formalin before μCT analysis (VIVA CT40, SCANO Medical, Bruttisellen, Switzerland). Scanning of the epiphyses was according to previously published procedures^6^. Microarchitecture reconstructions of the epiphyses were carried out and analyzed using the SCANCO software (SCANO Medical, Bruttisellen, Switzerland).

### Dynamic calcein labeling and histomorphometry

Mice were ip injected with calcein (20 mg/kg) at postnatal day (P) 22 and P26 and euthanized at P28. Femurs were fixed and processed as previously reported^12^. Calcein labeling was visualized with the Olympus BX60 fluorescence microscope (Olympus Corp). Bone formation and resorption parameters were measured using the OsteoMeasure software (Osteometrics Inc). BV/TV, BFR/TV, BFR/BV, BFR/BS, MAR, and Oc.S/BS were measured according to established methods^24^.

### Primary chondrocyte culture

Primary chondrocytes were isolated from SOCs of epiphyses and cultured as previously described^38^. Cells were grown in ascorbic acid-free αMEM medium containing 10% fetal bovine serum, penicillin (100 U/mL), and streptomycin (100 μg/mL) to 70% confluence followed by 24 hours serum free (0.1% BSA αMEM without ascorbic acid) treatment. Then the cells were treated with 500 μM DMOG (Cayman Chemical, Ann Arbor, MI) or vehicle control DMSO for 72 hours. The cells were then processed for RNA extraction.

### Quantitative RT-PCR

The epiphyses of tibias were dissected from 4 week old control and *Phd2*^*Col2-Cre*^ mice and snap frozen. RNA was extracted with Trizol reagent according to the manufacturer’s instructions (Invitrogen, Grand Island, NY). RNA samples were then reverse-transcribed into cDNA and followed by quantitative real time PCR as previously described^39^. The ∆∆CT method was used to calculate relative gene expression with *Ppia* used as an internal control^39^. Primer sequences are as following: *Hif-1α*, forward, 5′-TGACGGCGACATGGTTTACA-3′, reverse, 5′-AGCTCCGCTGTGTGTTTAGT-3′, *Hif-2α*, forward, 5′-TCATTGCTGTGGTGACCCAA-3′, reverse, 5′-GGTGGACACGTCTTTGCTCT-3′, *Vegfa*, forward, 5′-ATGCGGATCAAACCTCACCAAA-3′, reverse, 5′-TTCTGGCTTTGTTCTGTCTTTCTTT-3′, *Vegfb*, forward, 5′-ACGATGGCCTGGAATGTGTG-3′, reverse, 5′-GGTCTGCATTCACATTGGCTG-3′. Other primer sequences were reported in previous publications^11^,^12^.

### Statistics

Data were expressed as mean ± SEM (standard error of the mean) and were analyzed using Student’s T-test. *P* < 0.05 was considered statistically significant.

## Additional Information

**How to cite this article**: Cheng, S. *et al.* Prolyl Hydroxylase Domain-Containing Protein 2 (*Phd2*) Regulates Chondrocyte Differentiation and Secondary Ossification in Mice. *Sci. Rep.* **6**, 35748; doi: 10.1038/srep35748 (2016).
